# The Mental Effects of Alcohol
1Dr. Robert Jones, British Jour. of Inebriety, July 1904.


**Published:** 1904-07-30

**Authors:** 


### THE MENTAL EFFECTS OF ALCOHOL.[1]

The mental effects of alcohol differ according as they are the result of a single large dose or repeated smaller doses. The latter may have been kept well within the bounds of "moderation" and yet under the stress of shock or illness they may be responsible for evidences of mental disturbance. A tendency to delusions of persecution is very common in chronic alcoholics. Visual illusions, or delusions based upon them; delusions of the grandiose, boastful, or vainglorious order; and those of a suspicious and persecutory character are almost invariably caused directly or indirectly by alcohol. Alcoholic insanity may be hallucinatory or delusional in type, both varieties tending to terminate in dementia, which in some instances may be the primary form. The most characteristic symptom of alcoholic insanity is paramnesia—a failure of memory for recent events, a loss of the quality of nerve cells by which images of past sensations are retained and consequently a defect of the power of associating ideas. This condition—with its resulting confusion of memory—is largely responsible for the deliberate and shameless lying which usually distinguishes these patients. There is also a mental restlessness and wandering, the patient being confused as to time and place. Another peculiarity of chronic drinkers is the impulsiveness of all their reactions when these become excited. Hence such patients exhibit violent temper and unrestrained licence in action and speech. In one class the moral nature is much perverted but there still remains the knowledge of right and wrong and the capacity, under a sufficiently powerful stimulus, to abstain from alcohol.

[1] Dr. Robert Jones, British Jour. of Inebriety, July 1904.

